# Dutch workers’ attitudes towards having a coworker with mental health issues or illness: a latent class analysis

**DOI:** 10.3389/fpsyt.2023.1212568

**Published:** 2023-07-10

**Authors:** I. E. van Beukering, G. Sampogna, M. Bakker, M. C. W. Joosen, C. S. Dewa, J. van Weeghel, C. Henderson, E. P. M. Brouwers

**Affiliations:** ^1^Tranzo Scientific Center for Care and Wellbeing, Tilburg School of Social and Behavioral Sciences, Tilburg University, Tilburg, The Netherlands; ^2^The Netherlands Labour Authority, Den Haag, The Netherlands; ^3^Department of Psychiatry, University of Campania “L. Vanvitelli”, Naples, Italy; ^4^Department of Methodology and Statistics, Tilburg University, Tilburg, The Netherlands; ^5^Department of Psychiatry and Behavioral Sciences, University of California, Davis, CA, United States; ^6^Health Service and Population Research Department, King’s College London, Institute of Psychiatry, Psychology and Neuroscience, London, United Kingdom

**Keywords:** mental health, stigma, discrimination, workplace, coworker

## Abstract

**Introduction:**

Workplace mental health stigma is a major problem as it can lead to adverse occupational outcomes and reduced well-being. Although workplace climate is largely determined by managers and co-workers, the role of co-workers in workplace stigma is understudied. Therefore, the aims are: (1) to examine knowledge and attitudes towards having a coworker with Mental Health Issues or Illness (MHI), especially concerning the desire for social distance, (2) to identify distinct subgroups of workers based on their potential concerns towards having a coworker with MHI, and (3) to characterize these subgroups in terms of knowledge, attitudes, and background characteristics.

**Materials and methods:**

A cross-sectional survey was conducted among a nationally representative internet panel of 1,224 Dutch workers who had paid jobs and did not hold management positions. Descriptive statistics and a three-step approach Latent Class Analysis (LCA) were used to address the research aims.

**Results:**

Concerning the desire for social distance, 41.9% of Dutch workers indicated they did not want to have a close colleague with MHI, and 64.1% did not want to work for a higher-ranking manager who had MHI. In contrast however, most workers did not have negative experiences with interacting with coworkers with MHI (92.6%). Next, five distinct subgroups (SG) of workers were identified: two subgroups with few concerns towards having a coworker with MHI (SG1 and SG2; 51.8% of the respondents), one subgroup with average concerns (SG3; 22.7% of the respondents), and two subgroups with more concerns (SG4 and SG5; 25.6% of the respondents). Four out of five subgroups showed a high tendency towards the desire for social distance. Nevertheless, even in the subgroups with more concerns, (almost) half of the respondents were willing to learn more about how to best deal with coworkers with MHI. No significant differences were found between the subgroups on background characteristics.

**Discussion:**

The high tendency to the desire for social distance seems to contrast with the low number of respondents who personally had negative experiences with workers with MHI in the workplace. This suggests that the tendency to socially exclude this group was not based on their own experience. The finding that a large group of respondents indicated to want to learn more about how to deal with a co-worker with MHI is promising. Destigmatizing interventions in the workplace are needed in order to create more inclusive workplaces to improve sustained employment of people with MHI. These interventions should focus on increasing the knowledge of workers about how to best communicate and deal with coworkers with MHI, they do not need to differentiate in background variables of workers.

## 1. Introduction

Mental health stigma and discrimination in the workplace are a major problem for people with Mental Health Issues or Illness (MHI) (1, 2). The concept of stigma consists of three dimensions, problems with: knowledge (misinformation of ignorance), attitudes (prejudice), and behaviour (discrimination) (3). Stigma and discrimination can lead to adverse occupational outcomes and reduced well-being (4). Multiple large studies showed that people with MHI face stigma and discrimination at work. For instance, a study on discrimination among workers with major depressive disorder from 35 countries showed that 62.5% had anticipated and/or experienced discrimination at work (5). Also, a recent study showed that 64% of Dutch line managers were reluctant to hire a job applicant with a mental health issue (6). In addition, 68.4% of Dutch workers expected that disclosure during a temporary contract would decrease the chance that a contract would be renewed, and 56.6% expected that disclosure would lead to a diminished chance to be promoted to a higher position in the future (7). MHI affect a large part of the population, almost half of the adults (48%) in Netherlands (18–75 years old) has ever had one or more mental illnesses (8). As employment is important for health and well-being, workplace stigma and discrimination should be seen as a public health problem.

A recent systematic review showed that most publications on workplace stigma were focused on the roles of employers, while less is known about the roles of workers (9). However, workers have also found to be influential stakeholders with stigmatizing attitudes in the workplace (10). In an American study, workers were found to have concerns about competencies of coworkers with MHI and held unfavourable attitudes to work with a person with MHI (11). Furthermore, mental health stigma by workers can lead to bullying, harassment (11, 12) or social exclusion of people with MHI in the workplace (13). One study showed that examples of social exclusion (or more specific: the desire for social distance) are not wanting to working for or with people with MHI or excluding coworkers from social events at work (11).

Anti-stigma interventions can lead to more inclusive workplaces (14, 15). More specifically, these interventions can help to improve sustained employment of people with MHI by increasing workers’ knowledge and helping behaviours (15). Evaluating how processes of stigma are perpetuated in the workplace is essential for guiding the development of anti-stigma interventions (16). One hindering or perpetuating factor can be legislation (17), as are cultural differences (18), which may need to be taken into account when developing destigmatizing interventions. Anti-stigma interventions need to address the diverse needs of the stakeholders in the workplace (19). This way anti-stigma interventions can differentiate in the messages to each target group and therefore be more effective.

In Netherlands, several studies have shown that a variety of workplace stakeholders tend to have negative attitudes towards people with MHI, such as HR managers, line managers and coworkers (1, 6, 7, 19, 20). However, research on this topic is very scarce in Netherlands, especially on attitudes of workers in non-managerial positions, who often make up a large part of the social work environment. If we want to develop effective anti-stigma interventions, we first need to better understand the nature of those negative attitudes, and high quality research on the nature of these stakeholders’ concerns is needed. As such, we used a large and representative sample to study the following aims: (1) to examine knowledge and attitudes towards having a coworker with MHI, especially concerning the desire for social distance, (2) to identify distinct subgroups of workers based on their potential concerns towards having a coworker with MHI, and (3) to characterize these subgroups in terms of knowledge, attitudes, and background characteristics.

## 2. Materials and methods

### 2.1. Study design and participants

In February 2018, a cross-sectional survey was conducted among a nationally representative internet panel of Dutch workers. Data were collected among an existing panel (Longitudinal Internet Studies for Social Sciences, LISS) administered by CentERdata, which is a Dutch research institute specialized in data collection. The existing panel is a random and representative selection of 5,000 Dutch households (7,357 panel members). The questionnaires include domains like education, work, housing, time use, income, political views, values, and personalities. Three reminders were sent to panel members to increase the response rate, see www.lissdata.nl for more information.

The questionnaire was sent to 1,671 Dutch adults who participated in the panel, had paid jobs, and did not hold management positions. Ethical Approval was obtained by the Ethics Review Board of the School of Social and Behavioral Sciences of Tilburg University (registration number: RP193).

### 2.2. Research context

In Netherlands, the Gatekeeper Improvement Act and the Extended Payment of Income Act protect Dutch workers with disabilities. The Gatekeeper Improvement Act states that employers, the occupational physician (OP), and the worker, are responsible for the benefits and reintegration when workers are absent due to the occurrence of sickness. Workers have to meet with an OP when sickness absence occurs. The OP is responsible for performing an independent assessment which in cooperation with the worker leads to a reintegration plan. The Extended Payment of Income Act states that employers pay at least 70% of the income for the first 2 years of sickness absence. During these first 2 years employers are not allowed to fire the worker. There is no obligation to disclose MHI in the workplace.

### 2.3. Measures

At present, validated instruments to measure workplace stigma are scarce (4), and especially questionnaires focusing on workers’ attitudes are lacking. Therefore, a questionnaire was developed using a multistep procedure to address the aims of this study. To this end, first, the existing literature on the topic of workplace mental health stigma and discrimination was searched. The main topics of the questionnaire were identified based on the theoretical stigma model proposed by Thornicroft et al. (2007) (3). Specifically, the items on knowledge and attitudes were based on literature of workplace stakeholders’ knowledge, experience, and attitudes (21, 22). Second, the main topics found and the subsequent proposed items on the questionnaire were discussed with international experts in the field of mental health and stigma (both senior researchers and experts by experience) to modify and improve the questionnaire. Third, the questionnaire was pilot tested (e.g., concerning clarity) within the researchers’ network (*N* = 18) and improved where necessary based on the feedback received. This resulted in the final version of the questionnaire. The items used for this study can be found in Supplementary material Appendix 1. The following topics and items were addressed:

#### 2.3.1. Knowledge of and experience with MHI

Knowledge

- Respondents were asked to indicate the percentage of coworkers they thought will be affected in their organization by MHI during their working life. The ratio response category ranged from 0 to 100%. Due to the distribution of the variable, the variable was converted to 0 (<15%), 1 (15–25%), and 2 (>25%).
- A set of 15 items of different types of MHI, the respondents were asked about which type of MHI they think of when hearing or reading about ‘a coworker with MHI’. The response categories were 0 (no) and 1 (yes). The items were converted into three dichotomous variables. Association with stress, mental/emotional exhaustion, and burnout were merged into ‘association with work related mental disorders’ because these are the most important reasons for work related sickness absence in Netherlands (23). Association with anxiety, depression, addiction, and obsessive–compulsive disorder was converted into ‘association with common mental disorders’ because these disorders typically refer to common mental disorders. Association with other disorders like manic depressive/bipolar disorder, schizophrenia, post-traumatic stress disorder, borderline disorder, autism, psychosis, and eating disorder was merged into ‘association with other mental disorders’.

Personal experience

- As personal experience is a source of knowledge, it was assessed if respondents knew anyone with MHI (i.e., general familiarity with MHI). To assess general familiarity with MHI, the Level of Contact Report was used (24). Therefore, general familiarity with MHI was measured by a set of 9 items, these items represent different kinds of relationships. The nominal response categories were 0 (no) and 1 (yes). To create the general familiarity variable, the individual items were converted into the following categories: 0 (not familiar) if respondents did not know anyone who had or had had MHI; 1 (little familiar) when respondents indicated to know a family member who they had little contact with and/or an acquaintance and/or a coworker with who they did not work much with MHI, and 2 (very familiar) when respondents indicated to know a family member who they had a lot of contact with and/or a friend and/or a coworker with whom worked or had worked intensively with MHI.
- Respondents’ actual experience with interacting with coworkers with MHI in the workplace. The response categories of this single item were 1 (very unfavourable), 2 (rather unfavourable), 3 (neutral), 4 (rather favourable), 5 (very favourable), and 6 (not applicable/no experience with this). Personal experience was converted into the categories 0 (negative = very unfavourable/rather unfavourable), 1 (neutral = neutral), 2 (positive = rather favourable/very favourable), and 3 (none = not applicable/no experience with this).

#### 2.3.2. Attitudes towards a coworker with MHI

The desire for social distance

- A set of three items measured the desire for social distance, asking the respondent to what extent they would (1) want to have a coworker who had MHI (but who they would hardly work with), (2) want to have a coworker who had MHI (and who they would work with intensively), and (3) want to work for a higher-ranking manager with MHI. The response categories were 1 (absolutely not), 2 (rather not), 3 (neutral), 4 (would not mind), 5 (would like to very much), and 6 (not applicable). The response categories were merged into the categories 0 (no = absolutely not/rather not), 1 (neutral = neutral/not applicable), and 2 (yes = would not mind/would like to very much).

Willingness to support a coworker with MHI

- A set of six items measured willingness to support a coworker with MHI. Five items asking to what extent respondents agreed with the following statements: (1) I will free up extra time for a coworker with MHI so that we can talk about his/her MHI, (2) I am happy to offer practical support to a coworker with MHI, for example by temporarily taking on some of his/her work, (3) I find it hard to work with a coworker with MHI, (4) I would like to learn more about MHI in general, and (5) I would like to learn more about how I can best deal with coworkers with MHI. The response categories were 1 (strongly disagree), 2 (slightly disagree), 3 (neutral), 4 (slightly agree), and 5 (strongly agree). And additionally, one item (6) asking the respondent to what extent they would (1) want to know if a coworker has MHI. The response categories were 1 (absolutely not), 2 (rather not), 3 (neutral), 4 (would not mind), 5 (would like to very much), and 6 (not applicable). The response categories of the seven items were merged into the categories 0 (no = strongly disagree/slightly disagree/absolutely not/rather not), 1 (neutral = neutral/not applicable), and 2 (yes = slightly agree/strongly agree/would not mind/would like to very much).

Responsibility

- One item measured if workers agreed with the following statement: (1) people are mainly responsible for their MHI. This item was added because attribution of personal responsibility can contribute to stigmatizing attitudes (24). The response categories were 1 (absolutely not), 2 (rather not), 3 (neutral), 4 (would not mind), 5 (would like to very much), and 6 (not applicable). The response categories of the item were merged into the categories 0 (no = strongly disagree/slightly disagree/absolutely not/rather not), 1 (neutral = neutral/not applicable), and 2 (yes = slightly agree/strongly agree/would not mind/would like to very much).

Potential concerns

- A set of 15 items about potential concerns having a coworker with MHI, like: I need to take over his/her duties, I am not sure how to help this coworker, and the coworker will make mistakes. The items were categorized in concerns about incompetency, concerns about helping and dealing with coworker with MHI, and concerns about that the coworker with MHI will damage the workplace. The response categories were 0 (no) and 1 (yes). These specific items were derived from literature on beliefs as barriers to employment (25, 26).

#### 2.3.3. Background characteristics

- Several background characteristics were included in this study because they were expected to be associated with stigma, based on previous research (1, 27, 28). A personal characteristic, personally having (had) MHI, was included. This self-reported variable measured whether workers have (or have had) MHI, it was merged into 0 (no = no/I do not know) and 1 (yes = yes). Sociodemographic characteristics were added, i.e., age, gender, educational level, and marital status. Educational level was converted into the categories 0 (low = primary school/intermediate secondary), 1 (secondary = higher secondary education/preparatory university education) and 2 (high = higher education). Marital status was converted into the categories 0 (unmarried = separated/divorced/widow or widower/never been married) and 1 (married = married). The work-related characteristics included were gross income per month, type of industry, company size and workplace atmosphere. Type of industry was merged into 0 (private = agriculture, forestry, fishery, and hunting/mining/industrial production/utilities production, distribution, and trade/construction/retail trade/catering/transport, storage, and communication/finance/business services) and 1 (public = governments services, public administration, and mandatory social insurances/education/healthcare and welfare). Following the definition of the European Commission (Commission Recommendation 96/280/EC), company size was changed into 0 (small; ≤ 49 workers) and 1 (medium or large; ≥ 50 workers). The item ‘In my organization it is customary to look down on workers with MHI’ was converted into workplace atmosphere with the categories 0 (negative = slightly agree/strongly agree), 1 (neutral = neutral), and 2 (positive = strongly disagree/slightly disagree).

### 2.4. Statistical analyses

To address the first research aim (i.e., to examine Dutch workers’ knowledge and attitudes towards having a coworker with MHI, especially concerning the desire for social distance), descriptive statistics were used (means, standard deviations, and frequency table).

For the second and third research aim (i.e., to identify distinct subgroups of workers based on their concerns about having a coworker with MHI, and to characterize these subgroups in terms of knowledge, attitudes, and background characteristics), a three-step approach Latent Class Analysis (LCA) was used. In the first step, a latent class model was built using the 15 items that measured potential concerns. In the second step, workers were assigned to the different subgroups. In the last step, the characteristics (i.e., knowledge, experience, attitudes, and background characteristics) that were associated with the different subgroups were evaluated.

In the first step of the LCA, the most suitable number of subgroups (classes) was identified by using several fit indices. The three fit indices that were used were the Bayesian information criterion (BIC), the Akaike information criterion (AIC), and the Akaike information criterion with 3 as penalizing factor (AIC3). These indices weight the fit and parsimoniousness of the model (the best model has the lowest criteria), and the BIC is seen as the best performing goodness-of-fit indice (29). Furthermore, a bootstrap likelihood ratio test (BLRT) (30), was used to compare the different models. Lastly, the theoretical interpretation of the model was taken into account. The size of the smallest subgroup had to be at least 5% of the total sample size (31).

In the second step, the workers were assigned to the latent subgroup based on the posterior subgroup membership probability.

In the third step, to characterize the subgroups the associations with knowledge, attitudes, and background characteristics were examined. Some items contained missing data (i.e., company size and gross income per month), Latent GOLD’s imputation procedure helped imputing these missing data (32). Wald tests (*p* < 0.05) were used to examine whether there were differences between the subgroups.

SPSS version 24 was used for the data preparation and descriptive analyses and Latent GOLD 6.0 was used for the three-step approach LCA (33).

## 3. Results

A total of 1,224 respondents with paid jobs (and who were not working in management positions) filled out the questionnaire (response rate = 73.5%), 27.9% of the respondents indicated that they had a current or past MHI. Slightly more respondents were female (57.1%) and they had an average age of 44.6 years (SD = 12.1). More characteristics can be found in Table 1.

**Table 1:** Main features of the sample.

	*N*	%	M (SD)
*Personal characteristic*			
Current or past MHI	1,224		
Yes	342	27.9	
*Sociodemographic characteristics*			
Age (years)	1,224		44.6 (12.1)
Gender	1,224		
Male	525	42.9	
Female	699	57.1	
Educational level^*^	1,224		
Low	209	17.1	
Secondary	491	40.1	
High	524	42.8	
Marital status	1,224		
Unmarried	609	49.8	
Married	615	50.2	
*Work-related characteristics*			
Gross income per month (in Euros)	1,117		4,845 (2382)
Type of industry	974		
Private	546	56.1	
Public	428	43.9	
Company size	746		
Small (<=49 workers)	343	46.0	
Medium or large (>=50 workers)	403	54.0	
Workplace atmosphere	1,222		
Negative	135	11.0	
Neutral	381	31.2	
Positive	706	57.8	

^*^Highest level completed. Low = primary school. Intermediate secondary; secondary = higher secondary education. Preparatory university education; high = higher education.

### 3.1. Research aim 1: to examine Dutch workers’ knowledge and attitudes towards having a coworker with MHI

Table 2 shows that most of the respondents thought that less than 25% of the coworkers in their organization would be affected by MHI during their working life. Also, most respondents were thinking of work related disorders when they heard or read about ‘a coworker with MHI’ (71.7%) and fewer respondents thought of common or other (more severe) mental disorders. Three quarters of the respondents were familiar in general with MHI, and a quarter indicated that they did not know anyone who had or had had MHI (27.2%). Most respondents did not have negative personal experiences with interacting with coworkers with MHI (92.6%).

**Table 2:** Dutch workers’ knowledge and attitudes towards having a coworker with MHI (*N* = 1,224).

			%
Knowledge and experience	Knowledge	Estimated prevalence of MHI in organization	
		<15%	47.6
		15–25%	20.9
		25%>	31.5
		Association MHI: work related disorders	71.7
		Association MHI: common disorders	47.2
		Association MHI: other disorders	27.0
	Experience	General familiarity with MHI	
		Not familiar	27.2
		Little familiar	18.3
		Very familiar	54.5
		Personal experience with interacting with coworkers with MHI	
		Negative	7.4
		Neutral	29.4
		Positive	32.1
		None	31.1
Attitudes	Desire for social distance	Want to have a coworker with MHI, who you would hardly work with	
		No	21.9
		Neutral	66.2
		Yes	11.8
		Want to have a coworker with MHI, who you would work with intensively	
		No	41.9
		Neutral	46.0
		Yes	12.0
		Want to work for a higher-ranking manager with MHI	
		No	64.1
		Neutral	28.5
		Yes	7.4
	Willingness to support	Free up extra time for a coworker with MHI, so we can talk about his/her problems	
		No	11.7
		Neutral	27.9
		Yes	60.4
		I am happy to offer practical support to a coworker with MHI	
		No	13.3
		Neutral	28.1
		Yes	58.7
		I would like to learn more about MHI in general	
		No	26.1
		Neutral	39.3
		Yes	34.6
		I would like to learn more about how I can best deal with coworkers with MHI	
		No	18.2
		Neutral	32.2
		Yes	49.5
		Want to know if coworker has MHI	
		No	6.9
		Neutral	29.0
		Yes	64.1
		I do not find it hard to work with a coworker with MHI	
		No	25.5
		Neutral	39.0
		Yes	35.5
	Responsibility	People are mainly personally responsible for their MHI	
		No	22.6
		Neutral	23.8
		Yes	53.6
	Potential concerns	Concerns that coworker with MHI is incompetent	
		A1 The coworker cannot handle the work	45.0
		A2 You cannot count on this coworker	32.7
		A3 It will lead to long-term sickness absence	28.8
		A4 The coworker will make mistakes	24.2
		A5 The coworker has a lower work tempo	11.7
		Personal concerns about helping and dealing with coworker with MHI	
		B1 I am not sure how to help this coworker	38.0
		B2 I need to take over his/her work tasks	33.4
		B3 I am not sure how to deal with this coworker	30.2
		B4 I do not feel like talking about the coworker’s personal problems	8.9
		Concerns that the coworker with MHI will damage the workplace	
		C1 It will have a negative impact on the workplace atmosphere	32.9
		C2 It will lead to conflicts	24.4
		C3 The coworker poses a danger to him or herself or to others in the workplace	22.3
		C4 The coworker will cause damage to the relationships that are important to me/the organization	12.8
		C5 Talking about the problems will take up a lot of the other coworkers’ time	10.2
		C6 He/she can damage my or the organization’s reputation	6.1

Table 2 also shows the exploration of the attitudes towards having a coworker with MHI. Concerning the desire for social distance, a large proportion of respondents did not want to have a coworker with MHI if they have to work with them intensively (41.9%) or, a smaller proportion of workers, if they would hardly have to work with them (21.9%). The majority would not want to work for a higher-ranking manager who had MHI (64.1%). Though, the majority of the respondents would be willing to free up extra time for a coworker with MHI so that they can talk about his/her problems (60.4%) and is happy to offer practical support (58.7%). Almost half of the respondents would like to learn more about how they can best deal with coworkers with MHI (49.5%) or would like to learn more about MHI in general (34.6%). More than half of the respondents indicated that people are mainly personally responsible for their MHI (53.6%). Most frequently reported were the concerns that a coworker with MHI would not be able to handle the work (45.0%) and that respondents do not know how to help a coworker with MHI (38.0%). A small part of the respondents (14.8%) reported not having any concerns.

### 3.2. Research aim 2: to identify distinct subgroups of workers based on their concerns about having a coworker with MHI

Five distinct subgroups of workers can be distinguished based on the LCA. Table 3 shows the model fit indices for models with 1 to 10 classes. Both the BIC and the bootstrap likelihood ratio test suggest a five-class model, while the AIC suggests a 10-class model and the AIC3 a three-class model. Further inspection of the different models showed that the five-class model was both parsimonious and had a good theoretical interpretation. Therefore, the five-class solution was chosen.

**Table 3:** Fit indices for LCA.

	LL	BIC	AIC	AIC3	Npar	df	*p* value BLRT	Entropy *R*^2^
1-Cluster	−9439.000	18984.648	18908.000	18923.000	15	1,209	0.000	–
2-Cluster	−8737.807	17696.021	17537.615	17568.615	31	1,193	0.000	0.740
3-Cluster	−8604.513	17543.190	17303.026	17350.026	47	1,177	0.000	0.702
4-Cluster	−8498.522	17444.967	17123.045	17186.045	63	1,161	0.036	0.680
5-Cluster	−8428.960	17419.602	17015.921	17094.921	79	1,145	0.064	0.674
6-Cluster	−8392.822	17461.083	16975.645	17070.645	95	1,129	0.126	0.689
7-Cluster	−8358.960	17507.117	16939.920	17050.920	111	1,113	0.148	0.683
8-Cluster	−8339.418	17581.791	16932.837	17059.837	127	1,097	0.102	0.672
9-Cluster	−8321.791	17660.294	16929.582	17072.582	143	1,081	0.082	0.682
10-Cluster	−8305.313	17741.096	16928.626	17087.626	159	1,065	0.082	0.691

LL, log likelihood; BIC, Bayesian information criterion; AIC, Aikake information criterion; AIC3, Aikake information criterion 3; Npar, numbers of parameters; BLRT, bootstrap likelihood ratio test.

**Figure 1:**
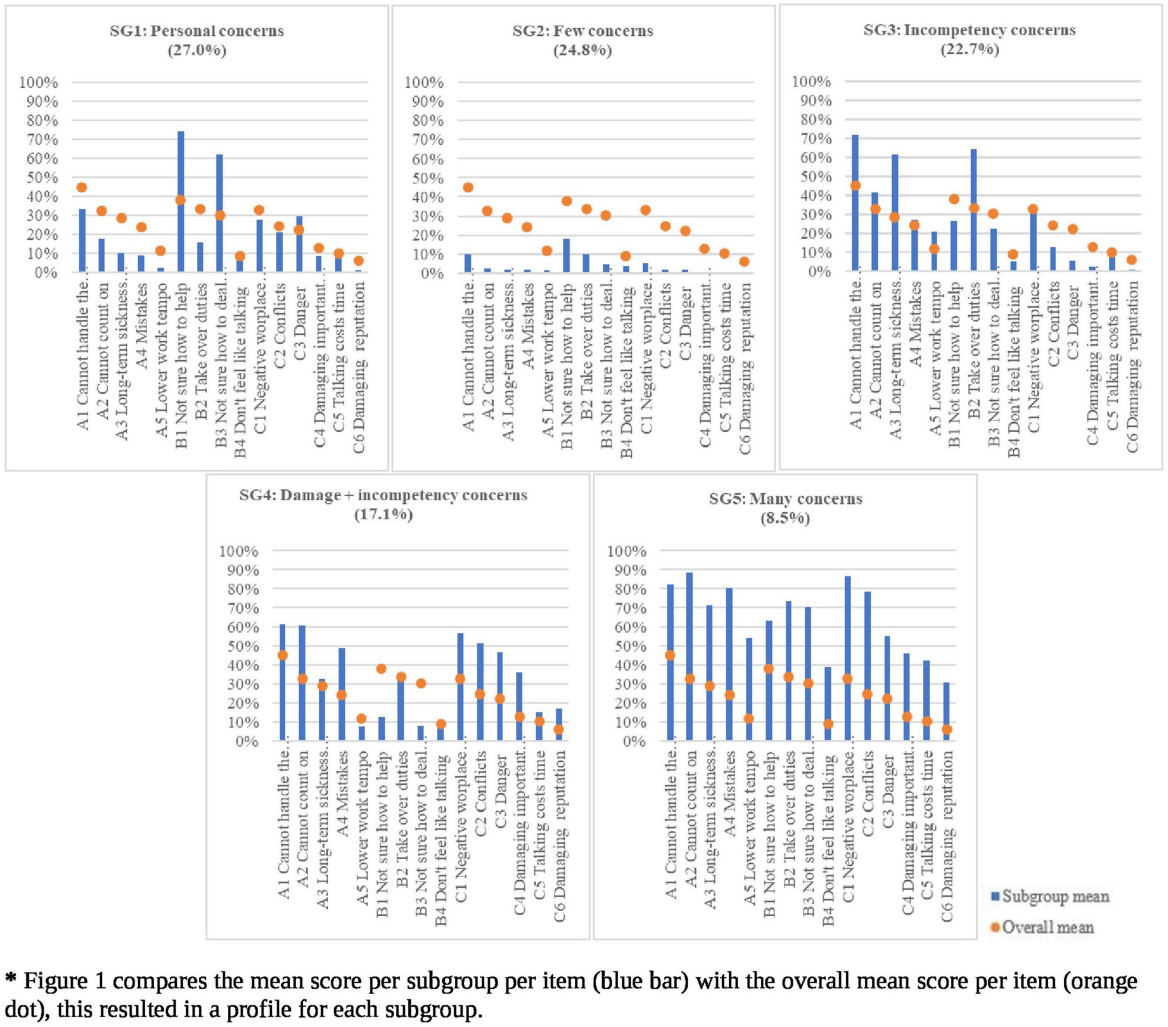
Profiles of the five subgroups based on potential concerns about having a coworker with MHI.

Figure 1 presents the five subgroups of respondents and their concerns about having a coworker with MHI. Significant differences between the subgroups were found on all the concerns. Respondents in the *few concerns* subgroup (SG2) have very few concerns about having a coworker with MHI (24.8% of the sample). Respondents in the *personal concerns* subgroup (SG1), which is the biggest subgroup (27.0% of the sample), have also low probabilities on most concerns, but are concerned about how they can help and deal with a coworker with MHI. Respondents in the *incompetency concerns* subgroup (SG3), have average probabilities on most concerns, but do have concerns that the coworker would be incompetent (22.7% of the sample). Respondents in the *damage and incompetency concerns* subgroup (SG4), have incompetency concerns and they are also concerned about damage to the workplace, but they have few concerns about how to help and deal with coworkers with MHI (17.1% of the sample). Respondents in the *many concerns* subgroup (SG5), the smallest subgroup (8.5% of the sample), have the highest probabilities on almost all concerns.

### 3.3. Research aim 3: to characterize these subgroups in terms of knowledge, experience, attitudes, and background characteristics

Respondents in the *few concerns* subgroup (SG2), i.e., with overall few concerns about having a coworker with MHI, scored the lowest on all the social distance items (ranging from 12.2% for not wanting to work with a coworker with MHI who they work intensively with to 28.8% not wanting to work for a higher ranking manager with MHI). SG2 contained the least respondents who were negative about wanting to have a coworker with MHI who they would have to work with intensively (12.0%), and the least respondents who were negative about wanting to work for a higher-ranking manager with MHI (28.8%). In SG2 the workers most often had no personal experience with interacting with coworkers with MHI (42.0%) (See Table 4).

**Table 4:** Characteristics of the subgroups in terms of knowledge, experience, attitudes, and background variables.

			SG1 Personal concerns (*N* = 330)	SG2 Few concerns (*N* = 304)	SG3 Incompetence concerns (*N* = 278)	SG4 Damage and incompetence concerns (*N* = 212)	SG5 Many concerns (*N* = 103)	*p*-value
Knowledge and experience	Knowledge	Estimated prevalence of MHI in organization						0.690
		<15%	54.3%	53.4%	35.3%	46.1%	45.5%	
		15–25%	21.9%	19.7%	23.7%	18.1%	19.8%	
		25%>	23.8%	26.9%	41.0%	35.8%	34.7%	
		Association MHI: work related disorders						0,000
		Yes	78.1%	53.7%	84.4%	66.8%	79.6%	
		Association MHI: common disorders						0.040
		Yes	51.4%	37.0%	37.4%	53.8%	77.1%	
		Association MHI: other disorders						0.000
		Yes	25.4%	18.7%	16.5%	43.1%	51.3%	
	Experience	General familiarity with MHI						0.170
		Not familiar	29.1%	41.9%	20.7%	13.1%	21.8%	
		Little familiar	19.0%	18.4%	20.1%	17.5%	10.6%	
		Very familiar	51.1%	38.6%	58.1%	68.6%	67.4%	
		Personal experience with interacting with coworkers with MHI						0.035
		Negative	4.5%	2.0%	8.5%	11.7%	20.5%	
		Neutral	22.8%	26.5%	33.5%	36.0%	34.6%	
		Positive	31.7%	29.5%	40.9%	30.2%	22.4%	
		None	41.1%	42.0%	17.1%	22.2%	22.5%	
Attitudes	Desire for social distance	Want to have a coworker with MHI, who you hardly work with						0.760
		No	16.0%	9.2%	23.8%	32.2%	40.2%	
		Neutral	70.5%	62.7%	62.8%	58.0%	50.6%	
		Yes	11.2%	14.2%	11.5%	8.6%	7.1%	
		Want to have a coworker with MHI, who you will work with intensively						0.000
		No	38.5%	12.0%	47.3%	57.7%	72.8%	
		Neutral	50.3%	58.7%	36.4%	36.6%	16.6%	
		Yes	9.2%	15.6%	15.1%	5.1%	9.6%	
		Want to work for a higher-ranking manager with MHI				0.014		
		No	67.2%	28.8%	70.2%	79.8%	77.8%	
		Neutral	27.1%	47.4%	18.6%	15.7%	15.9%	
		Yes	4.4%	10.9%	9.8%	3.9%	4.0%	
	Willingness to support	Free up extra time for a coworker with MHI, so we can talk about his/her problems						0.540
		No	8.3%	8.5%	13.5%	11.1%	28.4%	
		Neutral	25.8%	39.5%	26.0%	19.5%	22.3%	
		Yes	65.8%	52.0%	60.1%	69.0%	49.2%	
		I am happy to offer practical support to a coworker with MHI						0.580
		No	4.9%	13.0%	15.5%	14.7%	31.6%	
		Neutral	28.2%	38.6%	24.8%	20.8%	19.6%	
		Yes	66.8%	48.4%	59.3%	64.2%	48.6%	
		I would like to learn more about MHI in general						0.036
		No	19.6%	20.7%	32.5%	34.8%	27.2%	
		Neutral	41.5%	49.8%	31.4%	31.6%	37.5%	
		Yes	38.8%	29.5%	35.8%	33.2%	35.3%	
		I would like to learn more about how I can best deal with coworkers with MHI						0.000
		No	9.7%	19.3%	21.5%	22.2%	25.6%	
		Neutral	22.9%	47.0%	32.7%	30.4%	20.7%	
		Yes	67.4%	33.8%	45.5%	47.1%	53.6%	
		Want to know if coworker has MHI						0.006
		No	9.2%	6.1%	2.0%	7.8%	9.7%	
		Neutral	24.1%	44.3%	23.8%	18.8%	22.0%	
		Yes	65.6%	37.6%	73.4%	72.7%	66.1%	
		I do not find it hard to work with a coworker with MHI						0.000
		No	24.8%	11.0%	28.7%	26.4%	60.1%	
		Neutral	46.5%	42.4%	34.1%	35.6%	23.9%	
		Yes	28.6%	46.6%	36.9%	37.7%	15.9%	
	Responsibility	People are mainly personally responsible for their MHI						0.190
		No	18.4%	14.6%	24.5%	30.8%	37.1%	
		Neutral	20.1%	34.3%	22.1%	17.5%	22.0%	
		Yes	61.4%	51.1%	53.1%	51.4%	40.8%	
Background characteristics	^Personal^	Current or past MHI						0.110
		Yes	30.9%	25.1%	28.3%	29.8%	21.8%	
	Sociodemographic	Age (in years)						0.230
		Mean	44.0	46.4	43.0	46.9	41.2	
		Gender						0.250
		Male	50.9%	43.1%	32.6%	44.8%	40.5%	
		Female	49.1%	56.9%	67.4%	55.2%	59.5%	
		Educational level						0.110
		Low	13.6%	27.7%	11.7%	13.9%	18.1%	
		Secondary	34.3%	43.9%	36.8%	47.0%	42.7%	
		High	52.2%	28.4%	51.5%	39.1%	39.2%	
		Marital status						0.220
		Unmarried	53.2%	44.1%	50.0%	44.2%	66.4%	
		Married	46.8%	55.9%	50.0%	55.8%	33.6%	
	Work-related	Income (in Euros)						1.000
		Mean	4,872,76	4,592,50	5,077,28	4,962,71	4,628,07	
		Sector						0.074
		Private	52.6%	51.2%	32.5%	41.5%	38.5%	
		Public	26.1%	31.6%	45.6%	38.0%	38.5%	
		Company size						0.770
		Small	26.6%	28.6%	28.9%	29.6%	25.3%	
		Medium or large	35.4%	30.6%	32.2%	34.2%	31.1%	
		Workplace atmosphere						0.220
		Negative	12.2%	4.8%	14.8%	10.1%	17.4%	
		Neutral	27.1%	46.0%	19.0%	30.6%	33.8%	
		Positive	60.7%	49.2%	65.9%	58.9%	48.6%	

^*^Wald statistic. *p* < 0.05.

The respondents in the *personal concerns* subgroup (SG1), i.e., with overall few concerns but with concerns about how they can help and deal with a coworker with MHI, scored much higher on the social distance items compared to SG2 (ranging from 38.5% for not wanting to work with a coworker with MHI who they work intensively with to 67.2% not wanting to work for a higher ranking manager with MHI). In SG1 the respondents were slightly more often willing to like to learn more about MHI in general (38.8%) compared to other subgroups, but still, they rather preferred to learn more about how they could best deal with coworkers with MHI (67.4%). SG1 contained relatively more respondents with no personal experience with interacting with coworkers with MHI (41.1%) compared to the other subgroups.

The *incompetency concerns* subgroup (SG3), i.e., with average score on most concerns but with concerns about possible incompetency of the coworker with MHI, Compared to SG1 and SG2, contained more respondents who scored high on the social distance items (ranging from 47.3% for not wanting to work with a coworker with MHI who they work intensively with to 70.2% not wanting to work for a higher ranking manager with MHI). Almost half of SG3 would like to learn more about how they could best deal with coworkers with MHI (45.5%). SG3 differentiated from the other subgroups by containing the most respondents who had positive experiences with interacting with coworkers with MHI (40.9%).

The respondents in the *damage and incompetency concerns* subgroup (SG4), i.e., with slightly more concerns and specifically concerns on incompetency and also damage to themselves and the workplace, compared to the SG1, SG2, and SG3, contained relatively more respondents who scored high on the social distance items (ranging from 57.7% for not wanting to work with a coworker with MHI who they work intensively with to 79.8% not wanting to work for a higher ranking manager with MHI). Just like SG3, almost half of SG3 would like to learn more about how they could best deal with coworkers with MHI (47.1%). Respondents from SG4 were more likely to associate a coworker with MHI with other (more severe) disorders (43.1%) compared to the other subgroups.

The *many concerns* subgroup (SG5), i.e., with overall a lot of concerns, compared to the other subgroups, contained the most respondents who scored high on the social distance items (ranging from 72.8% for not wanting to work with a coworker with MHI who they work intensively with to 77.8% not wanting to work for a higher ranking manager with MHI). Respondents in this subgroup found it much harder to work with a coworker with MHI (60.1%), compared to the other subgroups. Around half of SG5 would like to learn more about how they could best deal with coworkers with MHI (53.6%). SG5 contained most respondents who associated a coworker with MHI other (more severe) disorders (51.3%), and the most respondents, but still a relatively small percentage, with a negative experience with interacting with coworkers with MHI (20.5%).

## 4. Discussion

The aims of this study were to examine (1) Dutch workers’ knowledge and attitudes towards having a coworker with MHI, especially concerning the desire for social distance, (2) to identify distinct subgroups of workers based on their potential concerns towards having a coworker with MHI, and (3) to characterize these subgroups in terms of knowledge, attitudes, and background characteristics. First, concerning the desire for social distance, nearly half of the respondents did not want to have a coworker with MHI who they would have to work with intensively and about two-thirds did not want to work for a higher-ranking manager who had MHI. Almost half of the respondents showed willingness to learn more about how to communicate and deal with coworkers with MHI. Very few workers had negative personal experiences with interacting with coworkers with MHI. The most frequently reported concern was that a coworker with MHI would not be able to handle the work. For the second research aim, five distinct subgroups of respondents were identified based on their concerns about having a coworker with MHI: two subgroups with few concerns (SG1 and SG2), one subgroup with average concerns (SG3), and two subgroups with more concerns (SG4 and SG5). Third, these subgroups were characterized by significant differences in knowledge, experience, and attitudes. Four out of five subgroups showed a high tendency towards the desire for social distance. Even in the subgroups with average and more concerns (almost) half of the respondents were willing to learn more about how to best deal with coworkers with MHI. The subgroups with more concerns contained most respondents who associated a coworker with MHI with other (more severe) disorders. No significant differences were found between the subgroups on background characteristics.

This study showed overall a high tendency towards the desire for social distance. When differentiated in subgroups, even higher rates were found for the subgroups with average or more concerns. This is worrying, and in line with previous research which reported that respondents did not want to work with or for people with MHI due to stigma (11). As 92.6% did not have negative personal experiences with interacting with coworkers with MHI, this tendency to the desire for social distance is not likely to be based on personal experiences. Our analyses showed that even in the subgroup with the most concerns (SG5) only 20.5% of the respondents had actual negative experiences with interacting with coworkers with MHI in the workplace. Moreover, the tendency towards exclusion without having negative experiences was also found in a study among Dutch line managers, where 64% was reluctant to hire a job applicant with a mental health issue, despite the fact that only 7% of them had actual negative experiences with such workers (6). Also, it is noteworthy that this present study showed that the tendency towards the desire for social distance is higher when respondents were asked about having to work for a higher-ranking manager with MHI compared to having to work with a coworker with MHI. A qualitative study also showed that negative disclosure outcomes were more likely to be expected for people with MHI in higher positions (1). More research is needed to understand this difference. The results concerning the high tendency towards the desire for social distance underline the importance of an adequately prepared disclosure decision. The high desire for social distance towards coworkers with MHI might also be partly due to the Dutch context. The Extended Payment of Income Act states that employers pay at least 70% of the income for the first 2 years of sickness absence. This might create an incentive for employers to be more careful during the hiring process, which can stimulate a culture of social distancing and exclusion.

To design an effective intervention it is important to understand what the focus needs to be, as stigma has three dimensions the focus can be on problems of: knowledge (misinformation or ignorance), attitudes (prejudice), and behaviour (discrimination) (21). Anti-stigma interventions in the workplace like increasing knowledge can lead to helping behaviour mediated by the change in attitudes, since these three dimensions are interrelated (15). This present study indicates that anti-stigma interventions in the workplace should focus on increasing knowledge, as there was a need among respondents to learn how to best deal with coworkers with MHI and to learn more about MHI in general. As the present study found no differences in background characteristics between the subgroups, this indicates that anti-stigma interventions in the workplace do not need to differentiate in background variables of workers.

### 4.1. Strengths and limitations

A strength of this study is the use of a large representative sample of Dutch workers. The workers were selected from population registers based on a true probability sample and participated anonymous to prevent the respondents’ possible tendency to underreport socially undesirable responses and overreport more socially desirable responses. Furthermore, this is one of the first datasets that focuses on workplace stigma in Netherlands which provides important new insights in the attitudes of workers. Another strength is that in this study coworkers were not seen as one homogenous group, but that heterogeneity was taken into account reflecting individual differences better which is needed for designing interventions. Latent Class Analysis, an increasingly popular method, is strong in identifying subgroups and it uses a model-based technique which enables researchers to have more flexibility and accuracy when looking into the subgroups and the associated variables (34). Although this study generated valuable insights, there are a few limitations. Self-reported data were used which were based on perceptions, rather than on actual behaviour. Nevertheless, perceptions have been linked to actual behaviour (35). Additionally, this study focused on concerns, which might reflect a more negative view of the reality because this study did not simultaneously focus on positive attitudes. Future studies should also focus on the positive attitudes in order to add more knowledge on both the positive and negative attitudes towards coworkers with MHI, because knowledge about such attitudes may also be helpful in designing interventions to create more inclusive workplaces.

## 5. Conclusion

This representative sample of Dutch workers showed a high tendency towards the desire for social distance of coworkers with MHI. As much as 41.9% did not want to have a coworker with MHI who they would work with intensively. The desire for social distance was even much higher towards managers with MHI: 64.1% did not want to work for a higher-ranking manager with MHI. Interestingly, despite these high percentages, over 92.6% of workers did not personally have negative experiences with interacting with coworkers with MHI. Workers differed in their concerns about having a coworker with MHI, five distinct subgroups were identified. Differences between these subgroups were found in knowledge, experience, and attitudes towards having a coworker with MHI. This study found that anti-stigma interventions in the workplace which focus on increasing knowledge are needed. This study found that anti-stigma interventions in the workplace which focus on increasing knowledge are needed, because (almost) half of the workers indicated they would like to learn more about MHI. These interventions should especially focus on increasing the knowledge of workers about how to best communicate and deal with coworkers with MHI and about MHI in general in order to create more inclusive workplaces to improve sustained employment of people with MHI.

## Data availability statement

The raw data supporting the conclusions of this article will be made available by the authors, without undue reservation.

## Ethics statement

The studies involving human participants were reviewed and approved by The Ethics Review Board of the School of Social and Behavioral Sciences of Tilburg University (registration number: RP606). The patients/participants provided their written informed consent to participate in this study.

## Author contributions

EB, MB, MJ, GS, and IB designed the study. MB assisted IB with the statistical analysis of the study. IB and GS wrote the manuscript. EB, MB, MJ, GS, CD, JW, and CH contributed to reviewing and revising of the manuscript. All authors contributed to the article and approved the submitted version.

## Funding

This study was funded by the Tilburg University Alumni Fund.

## Conflict of interest

The authors declare that the research was conducted in the absence of any commercial or financial relationships that could be construed as a potential conflict of interest.

## Publisher’s note

All claims expressed in this article are solely those of the authors and do not necessarily represent those of their affiliated organizations, or those of the publisher, the editors and the reviewers. Any product that may be evaluated in this article, or claim that may be made by its manufacturer, is not guaranteed or endorsed by the publisher.
