# Supplementary material for: Dutch workers’ attitudes towards having a coworker with mental health issues or illness: a latent class analysis
Source: Front Psychiatry. 2023 Jul 10;14:1212568. doi: 10.3389/fpsyt.2023.1212568 (PMC10365929; doi:10.3389/fpsyt.2023.1212568)
Supplement: Supplementary file 1 [file Table_1.DOCX]

Supplementary Material

**Dutch workers’ attitudes towards having a coworker with Mental Health Issues or Illness: a latent class analysis**

**Van Beukering I.E.*, Sampogna G., Bakker M., Joosen M.C.W., Dewa C.S., Van Weeghel J., Henderson C. & Brouwers E.P.M.**

*** Correspondence:** I.E. van Beukering: i.e.vanbeukering@tilburguniversity.edu

# Items used in questionnaire

|  | **Original language (Dutch)** | | **English** | |
| --- | --- | --- | --- | --- |
| Item | Vraag | Antwoordcategorie | Question | Response category |
| 1 | Hoeveel procent van alle werknemers in uw organisatie/bedrijf zal ooit psychische problemen krijgen tijdens hun werkende leven, denkt u? | 0…100 % | What percentage of employees in your  organization/company will be affected by mental health issues or illness during their working life, do you think? | 0…100 % |
| 2 | Waar denkt u aan als het gaat over ‘een werknemer met psychische problemen’? Ik denk **vooral** aan...  *Meerdere antwoorden mogelijk*  Angst  Depressie  Manische depressie/bipolaire stoornis  Stress  Verslaving  Obsessief-compulsieve stoornis (dwangstoornis)  Schizofrenie  Posttraumatische stressstoornis (PTSS)  Overspannenheid  Burn-out  Borderline stoornis  Autisme  Psychose  Eetstoornis | 0 Nee  1 Ja | What do you think of when you hear or read about ‘an employee with mental health issues or illness’? I **mainly** think of...  *Multiple answers possible*  Anxiety  Depression  Manic depressive/bipolar disorder  Stress  Addiction  Obsessive-compulsive disorder  Schizophrenia  Post-traumatic stress disorder (PTSS)  Mental/emotional exhaustion  Burnout  Borderline disorder  Autism  Psychosis  Eating disorder | 0 No  1 Yes |
| 3 | Kent u mensen die psychische problemen hebben (gehad)? Zo ja, wat is uw relatie met deze mensen?  *Meerdere antwoorden mogelijk*  Ik heb zelf psychische problemen (gehad)  Gezinslid en/of familielid waar ik veel contact mee heb  Gezinslid en/of familielid waar ik weinig contact mee heb  Vriend of vriendin  Kennis  Collega of werknemer met wie ik weinig samenwerk(te)  Collega of werknemer met wie ik veel samenwerk(te)  Anders, namelijk: …  Ik ken niemand die psychische problemen heeft (gehad) | 0 Nee  1 Ja | Do you know people who have (had) mental health issues or illness? If so, what is your relationship to these people?  *Multiple answers possible*  Personally have (had) mental health issues or illness  (Extended) family member who I have a lot of contact with  (Extended) family member who I have little contact with  A friend  An acquaintance  A colleague or employee with who I do not/did not work much  A colleague or employee with who I work/do not work intensively  Other relationship, namely: …  I do not know anyone who has (had) mental health issues or illness | 0 No  1 Yes |
| 4 | Hoe zijn over het algemeen uw ervaringen in het omgaan met collega’s met psychische problemen op de werkvloer? | 1 Heel slecht  2 Redelijk slecht  3 Neutraal  4 Redelijk goed  5 Heel goed  6 Niet van toepassing / geen ervaring | How would you describe your experience with dealing with employees with mental health  issues or illness in the workplace, generally speaking? | 1 Very unfavorable  2 Rather unfavorable  3 Neutral  4 Rather favorable  5 Very favorable  6 Not applicable / no experience |
| 5 | In hoeverre zou u...  Een collega willen krijgen die psychische problemen heeft, met wie u veel zal gaan samenwerken?  Een collega willen krijgen die psychische problemen heeft, met wie u zelf nauwelijks zal gaan samenwerken?  Voor een hogere leidinggevende (directeur) willen werken die psychische problemen heeft?  Het willen weten als een direct collega psychische problemen heeft? | 1 Absoluut niet graag  2 Niet zo graag  3 Neutraal  4 Best graag  5 Heel erg graag  6 Niet van toepassing | To what extent would you...  Want to have a colleague who has mental health issues or illness, and who you will  work with intensively?  Want to have a colleague who has mental health issues or illness, but who you will  hardly work with?  Want to work for a higher-ranking manager (director) who has mental health issues or illness?  Want to know if a direct colleague has mental health issues or illness? | 1 Absolutely not  2 Rather not  3 Neutral  4 Wouldn’t mind  5 Would like to very much  6 Not applicable |
| 6 | Geef aan in hoeverre u het eens bent met onderstaande stellingen.  *Als u nooit met een collega met psychische problemen hebt samengewerkt, dan graag invullen wat u zou doen/vinden.*  Voor een collega met psychische problemen maak ik extra tijd vrij om over zijn/haar problemen te praten.  Ik bied graag praktische ondersteuning aan een collega met psychische problemen, bijvoorbeeld door werk tijdelijk over te nemen.  Ik vind het moeilijk om samen te werken met een collega met psychische problemen.  Mensen zijn voornamelijk zelf verantwoordelijk voor hun psychische problemen.  Ik zou meer willen leren over psychische aandoeningen in het algemeen.  Ik zou meer willen leren over hoe ik het beste om kan gaan met collega’s met psychische problemen.  In mijn organisatie/bedrijf heerst een cultuur waarbij op werknemers met psychische problemen wordt neergekeken. | 1 Sterk mee oneens  2 Beetje mee oneens  3 Neutraal  4 Beetje mee eens  5 Sterk mee eens | To what extent do you agree with the statements below?  *If you never worked with a colleague with mental health issues* or illness*, then please indicate*  *what you would do/think*.  I will free up extra time for a colleague with mental health issues or illness so that we can talk about his/her problems.  I am happy to offer practical support to a colleague with mental health issues or illness, for example by temporarily taking on some of his/her work.  I find it hard to work with a colleague with mental health issues or illness.  People are mainly personally responsible for their mental health issues or illness.  I would like to learn more about mental health issues or illness in general.  I would like to learn more about how I can best deal with colleagues with mental health issues or illness.  In my organization/company, it is customary to look down on employees with mental health issues or illness. | 1 Strongly disagree  2 Slightly disagree  3 Neutral  4 Slightly agree  5 Strongly agree |
| 7 | Wat zijn uw eventuele **zorgen** over het hebben van een directe collega met psychische problemen?  Mijn (eventuele) zorgen zijn dat...  *Meerdere antwoorden mogelijk*  Ik zijn/haar werkzaamheden moet overnemen  Ik niet goed weet hoe ik de collega kan helpen  Ik niet goed weet hoe ik met de collega om moet gaan  Het een negatieve invloed heeft op de werksfeer  Hij/zij mijn imago of dat van de organisatie kan schaden  Je niet van de collega op aan kan  De collega fouten maakt  De collega een lager werktempo heeft  De collega schade aanricht in het contact met anderen die voor mijn bedrijf/organisatie belangrijk zijn (zoals klanten bij een bedrijf, of leerlingen bij een school)  De collega het werk niet aankan  De collega een gevaar oplevert voor zichzelf of anderen op het werk  Er conflicten ontstaan  Ik geen zin heb om te praten over de privéproblemen van de collega  Het zal leiden tot langdurig ziekteverzuim  Er veel tijd van andere collega’s gaat zitten in het praten over de problemen | 0 Nee  1 Ja | What are your potential **concerns** about having a direct colleague with mental health issues or illness?  My (potential) concerns are that...  *Multiple answers permitted*  I need to take over his/her duties  I’m not sure how to help this colleague  I’m not sure how to deal with this colleague  It will have a negative impact on the workplace atmosphere  He/she can damage my or the organization’s reputation  You cannot count on this colleague  The colleague will make mistakes  The colleague has a lower work tempo  The colleague will cause damage to relationships that are important to me/the  organization (such as company customers, or students at a school)  The colleague cannot handle the work  The colleague poses a danger to him or herself or to others in the workplace  It will lead to conflicts  I don’t feel like talking about the colleague’s personal problems  It will lead to long-term sickness absence  Talking about the problems will take up a lot of the other colleagues’ time | 0 No  1 Yes |
| 8 | Hebt u zelf psychische problemen (gehad)? | 1 Ja  2 Nee  3 Ik weet het niet | Do you have (or have you had) mental health issues or illness, personally? | 1 Yes  2 No  3 I don’t know |
| 9 | Leeftijd van lid huishouden |  | Age of the household member |  |
| 10 | Geslacht | 1 Man  2 Vrouw | Gender | 1 Male  2 Female |
| 11 | Opleiding in CBS-categorieën | 1 Basisonderwijs  2 VMBO  3 HAVO/WVO  4 MBO  5 HBO  6 WO | Highest level of education in CBS (Statistics Netherlands) categories | 1 Primary school  2 VMBO (intermediate secondary education, US: junior high school)  3 HAVO/VWO (higher secondary education/preparatory university  education, US: senior high school)  4 MBO (intermediate vocational education, US: junior college)  5 HBO (higher vocational education, US: college)  6 WO (university) |
| 12 | Burgerlijke staat | 1 Gehuwd  2 Gescheiden van tafel en bed  3 Gescheiden  4 Weduwe of weduwnaar  5 Nooit getrouwd | Marital status | 1 Married  2 Separated  3 Divorced  4 Widow or widower  5 Never been married |
| 13 | Bruto maandinkomen huishouden in euro’s  *Geïmputeerde maandinkomens (brutoink_f) van alle huishoudleden bij elkaar opgeteld.* |  | Gross household income in Euros  *Imputed monthly income (brutoink_f) of all household members combined.* |  |
| 14 | In welke branche bent (/was) u werkzaam (in uw laatste werkkring)? | 1 Landbouw, jacht, bosbouw, visserij  2 Winning van delfstoffen  3 Industrie  4 Productie en distributie van en handel in elektriciteit, aardgas,  stroom en water  5 Bouwnijverheid  6 Handel (incl. reparatie van consumentenartikelen)  7 Horeca  8 Vervoer, opslag en communicatie  9 Financiële instellingen  10 Zakelijke dienstverlening (incl. onroerend goed, verhuur van roerende goederen)  11 Overheidsdiensten, openbaar bestuur en verplichte sociale  verzekeringen  12 Onderwijs  13 Gezondheids- en welzijnszorg  14 Milieudienstverlening, cultuur, recreatie en overige dienstverlening  15 Overig |  | 1 Agriculture, hunting, forestry, fishery  2 Mining  3 Industrial production  4 Utilities production, distribution, trade  5 Construction  6 Retail trade  7 Catering  8 Transport, storage, and communication  9 Finance  10 Business services  11 Governments services, public administration, and mandatory social insurances  12 Education  13 Healthcare and welfare  14 Environmental services, culture, recreation and other services |
| 15 | Hoeveel mensen werken (/werkten) (ongeveer) in de vestiging waar u (vooral) werkzaam bent (/was)? | -9 Ik weet het niet | Approximately, how many people work (were working) at the  location where you (mainly) work (/worked)? | -9 I don't know |
